# The effectiveness of Baduanjin exercise for hypertension: a systematic review and meta-analysis of randomized controlled trials

**DOI:** 10.1186/s12906-020-03098-w

**Published:** 2020-10-08

**Authors:** Bao-yi Shao, Xia-tian Zhang, Robin W. M. Vernooij, Qiu-yi Lv, Yao-yang Hou, Qi Bao, Li-xing Lao, Jian-ping Liu, Ying Zhang, Gordon H. Guyatt

**Affiliations:** 1 Li Ka Shing Faculty of Medicine, The University of Hong Kong, Hong Kong, China; 2 School of Mathematics Sciences, University of Southampton, Southampton, SO17 1BJ UK; 3 Department of Nephrology and Hypertension, University Medical Center Utrecht, Utrecht University, Utrecht, The Netherlands; 4 Julius Center for Health Sciences and Primary Care, University Medical Center Utrecht, Utrecht University, Utrecht, The Netherlands; 5 The First Affiliated Dongzhimen Hospital, Beijing University of Chinese Medicine, Beijing, China; 6 Guang’anmen Hospital of China Academy of Chinese Medical Sciences, Beijing, China; 7 Virginia University of Integrative Medicine, Fairfax, VA USA; 8 Center for Evidence-based Chinese Medicine, Beijing University of Chinese Medicine, Beijing, China; 9 Department of Health Research Methods, Evidence and Impact, McMaster University, Hamilton, Canada

**Keywords:** Baduanjin, Hypertension, Systematic review, Meta-analysis, Randomized controlled trials

## Abstract

**Background:**

Hypertension, a major risk factor of cardiovascular mortality, is a critical issue for public health. Although Baduanjin (Eight Brocades, EB), a traditional Chinese exercise, might influence blood pressure, glucose, and lipid status, the magnitude of true effects and subgroup differences remains unclear. Therefore, we performed a systematic review of relevant randomized controlled trials (RCTs) to evaluate the effect of EB on patient-important outcomes.

**Methods:**

We systematically searched PubMed, the Cochrane Library, Web of Science, and Chinese databases since inception until March 30, 2020. Meta-analysis was carried out using “meta” package in R 3.4.3 software. A prespecified subgroup analysis was done according to the type of comparisons between groups, and the credibility of significant subgroup effects (*P* < 0.05) were accessed using a five-criteria list. A GRADE evidence profile was constructed to illustrate the certainty of evidence.

**Results:**

Our meta-analysis, including 14 eligible trials with 1058 patients, showed that compared with routine treatment or health education as control groups, the mean difference (MD) in systolic blood pressure (SBP) of the EB groups was − 8.52 mmHg (95%CI:[− 10.65, − 6.40], *P* < 0.01) and diastolic blood pressure (DBP) was − 4.65 mmHg (95%CI: [− 6.55, − 2.74], P < 0.01). For blood pressure, the evidence was, however, of low certainty because of risk of bias and inconsistency, and for the outcomes of most interest to patients (cardiovascular morbidity and mortality directly), of very low certainty (measurement of surrogate only). Subgroup analysis showed there was no significant interaction effect between different type of comparisons (SBP *P* = 0.15; DBP *P* = 0.37), so it could be easily attributed to chance.

**Conclusion:**

Regularly EB exercising may be helpful to control blood pressure, but the evidence is only low certainty for blood pressure and very low certainty for cardiovascular morbidity and mortality. Rigorously designed RCTs that carry out longer follow-up and address patient-important outcomes remain warranted.

**Trial registration:**

PROSPERO Registration number: CRD42018095854.

## Background

Hypertension is one of the most prevalent conditions in the world and is commonly regarded as one of the main contributors to cardiovascular morbidity [1]*.* High blood pressure (HBP) affects over 1.39 billion people around the world and could lead to an estimated 9.4 million deaths per year, which makes hypertension one of the most serious chronic problems threatening public health [2–4]*.* As a leading risk factor for fatal cardiovascular disease, hypertension is associated with increased risk of myocardial infarction (MI), stroke, peripheral artery disease (PAD), end-stage renal disease [5]*,* and premature death [6], which greatly affects the quality of life and brings significant economic burdens to patients and their families [6]*.*

Modern therapies for hypertension include single or multiple pharmacological treatments as well as lifestyle modification [7]. Due to different socioeconomic and medical environmental factors, some patients, particularly those in developing countries, often show low adherence to antihypertensive therapy, which greatly reduces treatment efficacy [6]*.* Different classes of antihypertensive drugs may lead to different side effects [8]. In contrast to pharmacological treatments, United States guidelines indicate that as a nonpharmacological intervention, physical activity with systematic exercise plans is the recommended first line therapy to control blood pressure. Guidelines for hypertension in Canada and China also point out the importance of physical exercise as a health behavior management tactic for the prevention and treatment of hypertension [9–11]*.* Nevertheless, despite a general recognition to the positive effects of physical exercises on treating hypertension, due to variations in clinical evidence it is difficult to determine a standardized physical activity regimen [12]*.* Among the available options, however, aerobic is one kind of recommended physical activities worldwide.

Baduanjin qigong, a type of low-intensity aerobic exercise that enjoys a long history in traditional Chinese exercise, may have a positive impact on treating hypertension and metabolic diseases [13]*.* Baduanjin is a set of independent and complete fitness skills, consisting of eight decomposition actions, with each action having its own efficacy corresponding to a certain part of body, and together adjusting the whole body through each part. Ancient Chinese compared this set of movements to “Brocade”, representing beauty and luxury, and therefore Baduanjin is called Eight Brocades (EB).

Results from clinical and epidemiological studies have suggested that the long-term practice of EB may improve physical fitness and mental health, and have a positive impact on conditions such as ischemic stroke, knee osteoarthritis, hyperlipidemia, diabetes, chronic obstructive pulmonary disease and hypertension [14–19]*.* However, systematic summaries of the latest evidence regarding the impact of EB on blood pressure, including relevant subgroup differences have not yet been conducted. Therefore, we conducted a more rigorous and complete systematic review addressing how EB, on top of health education and routine treatment, may improve the effectiveness to modify blood pressure. Because diabetes and dyslipidemia are also very common and EB may impact on these conditions, as a secondary goal we examined the effect of EB on these outcomes.

## Methods

### Search strategies

We systematically searched the following databases since inception until March 30, 2020: PubMed, the Cochrane Library, Web of Science, Scopus, and Chinese databases including China National Knowledge Infrastructure Databases (CNKI), Chinese Biomedical Database (CBM), VIP, and Wan Fang Database. Additional file 1 presents the search strategies used in each database.

### Inclusion criteria

1. Type of study: We included randomized controlled trials (RCTs) reported in English or Chinese assessing EB for hypertension.
2. Type of participant: Patients with the following definition of HBP were included [20, 21]: systolic blood pressure (SBP) ≥140 mm Hg or diastolic blood pressure (DBP) ≥90 mm Hg; or a previous physician diagnosis of hypertension. We placed no restrictions on age, sex, race, or duration of hypertension.
3. Types of intervention: EB alone or EB combined with either routine treatment (like antihypertensive drugs, Chinese herbal decoctions etc.) or health education were considered as interventions. Exercise sessions were at least 4 weeks in duration. There was no limitation on the type of EB and the settings.
4. Types of control group: Health education, routine treatments like antihypertensive drugs or Chinese herbal decoctions etc. Interventions other than EB were the same in intervention and control groups.
5. Outcomes: The primary outcome measures were the SBP and DBP at the end of follow-up. The secondary outcome measures were glucose (GLU), serum total triglyceride (TG), serum total cholesterol (TC), high density lipoprotein cholesterol (HDL-C) and low density lipoprotein cholesterol (LDL- C).

The following studies were excluded: (a) Studies that lacked data for outcome evaluation even after contacting authors; (b) Besides antihypertensive drugs, Chinese decoctions, or health education, studies in which EB was also combined with other kind of therapies like acupuncture, sitting; (c) Studies that examined a special population of hypertension, such as those with severe hypertension (SBP ≥180 mm Hg or DBP ≥110 mm Hg), pregnancy-related hypertension or adolescent hypertension.

### Data extraction

Teams of two reviewers screened the titles and abstracts independently and obtained full-text articles of studies that potentially met eligibility criteria. A third reviewer (YZ) was responsible for adjudicating discrepancies between the reviewers. The two independent reviewers extracted the data from eligible studies and entered it into EpiData 3.2 (The EpiData Association, Odense, Denmark) including:(1) title, authors, publication year, study location and setting; (2) participants’ age, gender, duration of hypertension, diagnostic criteria, SBP, DBP, GLU, TG, TC, HDL-C and LDL-C at baseline and after treatment; (3) interventions, type of EB, controls, treatment duration, and risk of bias (ROB) assessment. Disagreements were resolved through discussion with the third reviewer.

### Certainty of evidence assessment

Teams of reviewers independently addressed the risk of bias (ROB) using the modified Cochrane ROB tool that includes response options of “definitely or probably yes (assigned a low risk of bias and showed green in the ROB figure)” or “definitely or probably no (assigned a high risk of bias and showed red in the ROB figure)” [22–24]. A GRADE evidence profile was constructed to illustrate the certainty of evidence. For the included RCTs, we rated down the certainty of evidence due to serious ROB, imprecision, inconsistency, indirectness and publication bias [25]*.*

### Statistical analysis

Meta-analysis including subgroup’s analysis was carried out using “meta” package in R 3.4.3 (The R Foundation for Statistical Computing, Vienna, Austria). For continuous variables, a mean difference (MD) with a corresponding 95% CI was calculated by using random effect models. Funnel plots, along with Begg’s and Egger’s test were used to address potential publication bias, were constructed when the number of included studies was more than 10.

### Subgroup analysis

A prespecified subgroup analysis was done according to the type of comparisons between groups when there were two or more studies in a given subgroup. We hypothesized that the difference between EB plus routine treatment and routine treatment alone would be smaller than that between EB plus health education and health education alone. Tests of interaction were conducted to establish whether the subgroups differed significantly from one another. We assessed the credibility of significant subgroup effects (*P* < 0.05) using a five-criteria list [26] (Additional file 2).

## Results

### Study selection

**Fig. 1:**
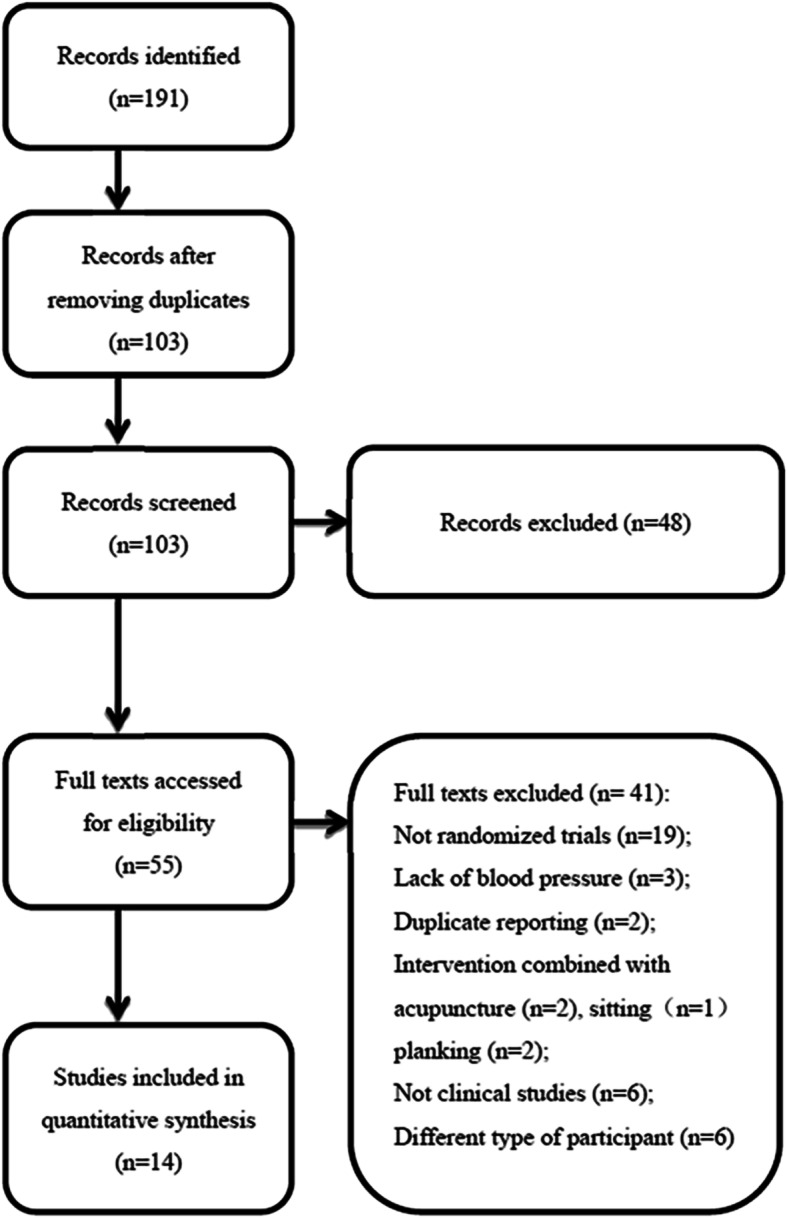
Study selection flow diagram

The initial database search identified 191 references. After excluding duplicated or irrelevant articles, 55 articles proved potentially eligible, of which 41 studies were excluded on full text review because they met one or more of the following criteria: they were not clinical trial studies (e.g. science articles from newspapers); patients were not randomized; duplicate reports; participants’ blood pressure (BP) were lower than the minimum value of our inclusion criteria; participants lacked BP values as observation index at baseline; or their treatment was combined with other therapies such as ear acupuncture, acupuncture or sitting. Finally, 14 papers proved eligible [27–40] (Fig. 1).

### Description of studies

Table 1 summarizes the main characteristics of the included studies. In total, 14 studies, published from 2010 to 2019, included 1058 patients in mainland China, with three studies conducted in the North of China and the rest in the South. With regard to the definition of hypertension in these studies, six applied the criteria in 2010 China Guideline [20], two studies used the criteria in 2005 China Guideline, three referred to 1999 WHO Guideline [21]; all met our inclusion criteria mentioned above. In terms of types of EB, five studies followed the standard exercise issued by the General Administration of Sport of China in 2003. Six studies failed to specify the types of EB; we assumed them as compliant with the 2003 version, as most types of EB share the same rationale and procedures. One article conducted the self-made “antihypertension EB” for intervention [28] and the other two studies evaluated the effectiveness of sitting EB [29, 38]*.* The intervention frequency of EB was twice a day in five studies, and four to five times a week for the remainder. For intervention duration per week, eight studies specified EB of more than 150 min per week while six studies applied less. Most of the studies were two-armed parallel; two studies included three groups, in which case we excluded the third group as it was another intervention group rather than a control group. No study reported adverse events.

**Table 1:** Characteristics of included studies

ID	Year	Stage	Disease course (years)	Male/ Female	Age (EG/CG)		Intervention(s) of the EG	Intervention(s) of the CG	Details of ①/ ②	Outcomes	Duration (days)	Number of subjects (EG/CG)
EB + ① vs. ①												
Pan	2010	I	EG:1.50 ± 1.20 CG:1.70 ± 0.80	EG:14/10 CG:13/11	62.10 ± 5.80	61.40 ± 7.10	EB + ①	①	Thiazide diuretics, Gastrodia and Uncaria Decoction	SBP, DBP, GLU, TG, TC, HDL-C, Insulin	168	24/24
Chen	2012	I, II	EG:10 ± 8 CG:11 ± 7	EG:25/15 CG:23/17	59 ± 6	60 ± 5	EB + ①	①	Nifedipine extended-release tablets 10-20 mg/time, 2times/d	SBP, DBP, Serum hs-CRP	168	40/40
Chen	2013	I	EG:9.13 ± 3.69 CG:8.30 ± 4.36	EG:13/14 CG:16/12	70.06 ± 3.51	69.23 ± 3.72	EB + ①	①	Anneizhen 5 mg or Norvasc 5 mg or Telmisartan 80 mg, 1 time/d	SBP, DBP, Serum NO, Plasma ET-1	84	30/30
Liao	2013	I, II	EG:4.80 ± 2.10 CG:3.90 ± 3.10	EG:38/32 CG:36/34	60.50 ± 11.80	62.70 ± 9.50	EB + ①	①	Walking 40 min + Amlodipine 5 mg, 1 time/d	SBP, DBP, FBG, TC, TG, BMI, HbA1c, Waist, Insulin	180	70/70
Liang	2014	I, II	EG:4.30 ± 3.00 CG:4.70 ± 3.20	EG:20/10 CG:18/12	54.80 ± 7.60	55.70 ± 8.80	EB + ①	①	NR	SDP, DBP, TC, TG, HDL-C, LDL-C	180	30/30
Yang	2014	I, II	NR	EG:19/16 CG:13/22	60.07 ± 5.84	60.60 ± 7.37	EB + ①	①	NR	SBP, DBP, SF-36, Heart Rate, Respiration	168	35/35
He	2015	I	EG:8.23 ± 3.73 CG:8.51 ± 3.42	EG:22/20 CG:23/19	68.51 ± 2.97	69.24 ± 2.45	EB + ①	①	NR	SBP, DBP	90	42/42
Chen	2016	I	EG:8.12 ± 3.53 CG:8.61 ± 3.32	EG:15/13 CG:14/14	69.98 ± 3.31	70.29 ± 1.77	EB + ①	①	NR	SBP, DBP	84	28/28
Liang	2016	Isolated systolic hypertension	EG:9.3 ± 2.6 CG:11.9 ± 5.8	EG:17/13 CG:16/14	68.1 ± 10.1	70.5 ± 10.2	EB + ①	①	Amlodipine 5 mg, 1 time/d (add Valsartan 80 mg, 1time/d, when necessary)	SBP, DBP, Self-made quality of life scale	90	30/30
Lin	2017	I	NR	62/54	58 ± 7.48		EB + ①	①	Amlodipine 5 mg or Telmisartan 80 mg, 1time/d	SBP, DBP, Heart Rate, NO, ET-1	180	58/58
EB + ②. vs. ②												
Dong	2016	I	0.42 ± 0.08	34/26	51.40 ± 4.20		EB + ②	②	Routine health education	DBP, SBP	60	30/30
Yu	2013	I	NR	NR	NR	NR	EB + ②	②	Intensive education per 2 months during the treatment period	SBP, DBP, BMI, WHR	360	52/52
Shi	2017	I	EG:2.55 ± 1.36 CG:2.67 ± 1.25	EG:19/11 CG1:18/12	42.65 ± 9.85	41.58 ± 9.12	EB + ②	②	Low-salt and low-fat diet education	SBP, DBP	180	30/30
Li	2019	I	EG ≤ 5,23>5,6CG: ≤5,20;>5,7	EG:6/23 CG:6/21	57.41 ± 3.38	55.81 ± 4.09	EB + ②	②	Diet education	FBG, SBP, DBP, HbA1C	360	30/30

Abbreviations: EG = Experimental Group; CG = Control Group; NR = Not Reported; EB = Eight Brocades = Baduanjin Qigong; I = hypertension of type I; II = hypertension of type II; ① = Routine treatment; ②Health education; SBP = Systolic Blood Pressure; DBP = Diastolic Blood Pressure GLU = Glucose; TG = Serum Total Triglyceride, TC = Serum Total Cholesterol; HDL-C = High Density Lipoprotein Cholesterol; LDL-C = Low Density Lipoprotein Cholesterol; Anneizhen = Domestic produced amlodipine besylate tablets. NR = Not Reported

### Certainty of evidence

**Fig. 2:**
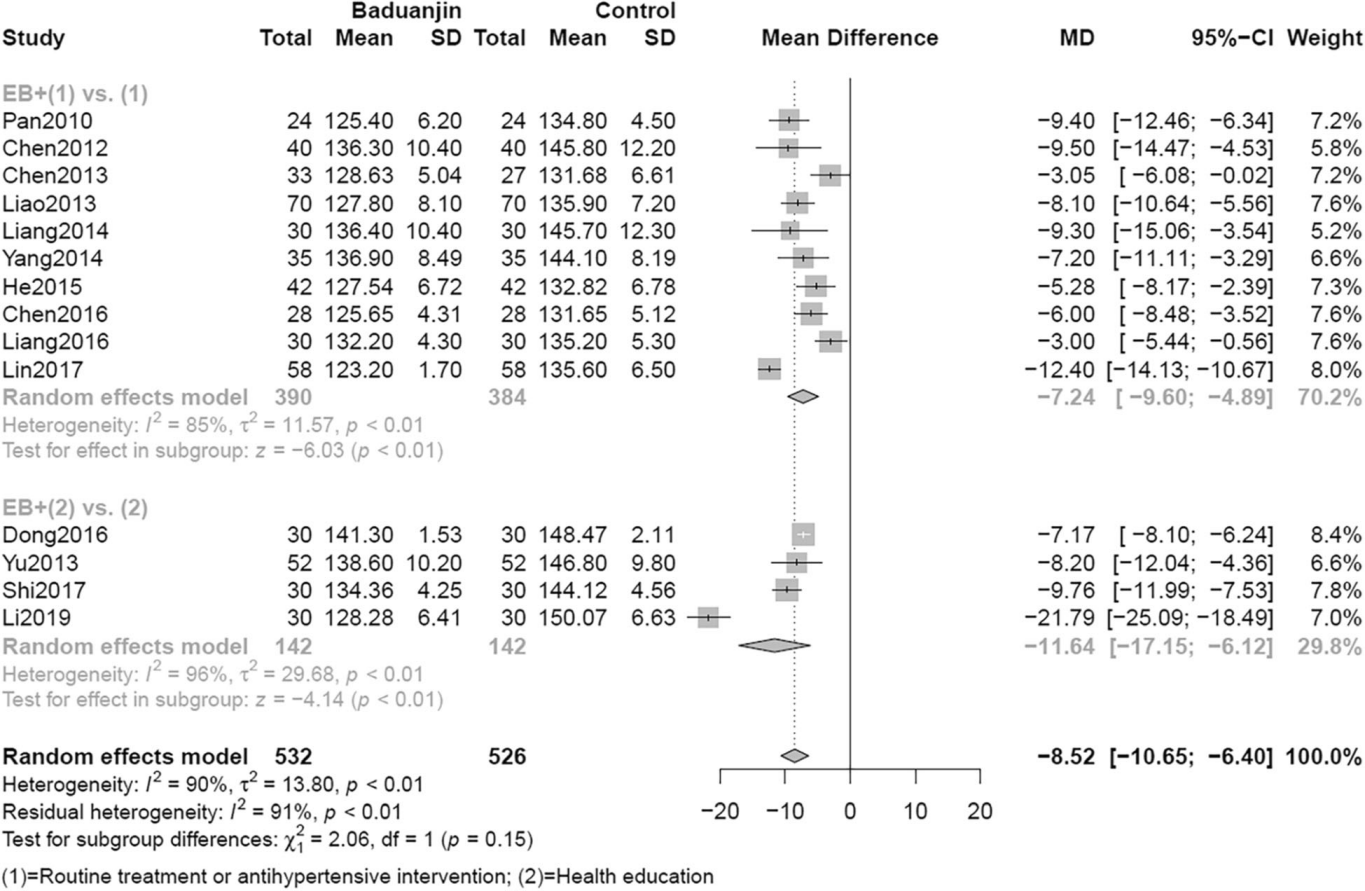
The funnel plot on SBP. SBP: Systolic Blood Pressure

**Fig. 3:**
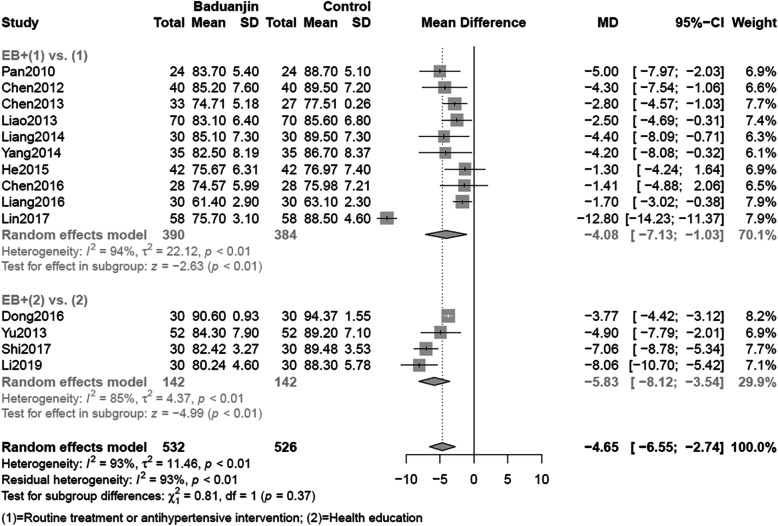
The funnel plot on DBP. DBP: Diastolic Blood Pressure

Table 2 presents the details of the risk of bias (ROB) evaluation. Of the 14 included studies, the randomization procedure was reported in adequate detail in seven studies, but all failed to report their methods for sequence generating. No study clearly reported the allocation concealment or blinding procedure, but reports made it evident that there was no blinding of participants or clinicians. Three studies reported missing data but did not use any imputation during analyzing data. As the missing data did not exceed 10% of the total sample size, we judged the risk of bias as probably low for that item. All studies had a low ROB in selective outcome reporting. Publication bias was evaluated visually by funnel plot (Figs. 2 and 3). From the distribution of scatterplots, which indicates a relationship between treatment effect estimates and study precision, small study effects may not exist. Begg’s (z = − 0.71, *P* = 0.48 for SBP; z = − 0.27, *P* = 0.78 for DBP) and Egger’s test (t = − 0.47, *P* = 0.64 for SBP; t = − 0.22, *P* = 0.83 for DBP) also did not suggest asymmetry in funnel plot. Therefore, publication bias was not leading us to rate down the level of certainty for the SBP and DBP outcomes. Publication bias remains suspect for other outcomes as only a few studies are available and all of them are small in size. Table 3 presents the GRADE evidence profile that shows that we rated down for ROB, inconsistency, and indirectness (we were interested in patient-important outcomes and all studies reported only on surrogates) for all outcomes.

**Table 2:**
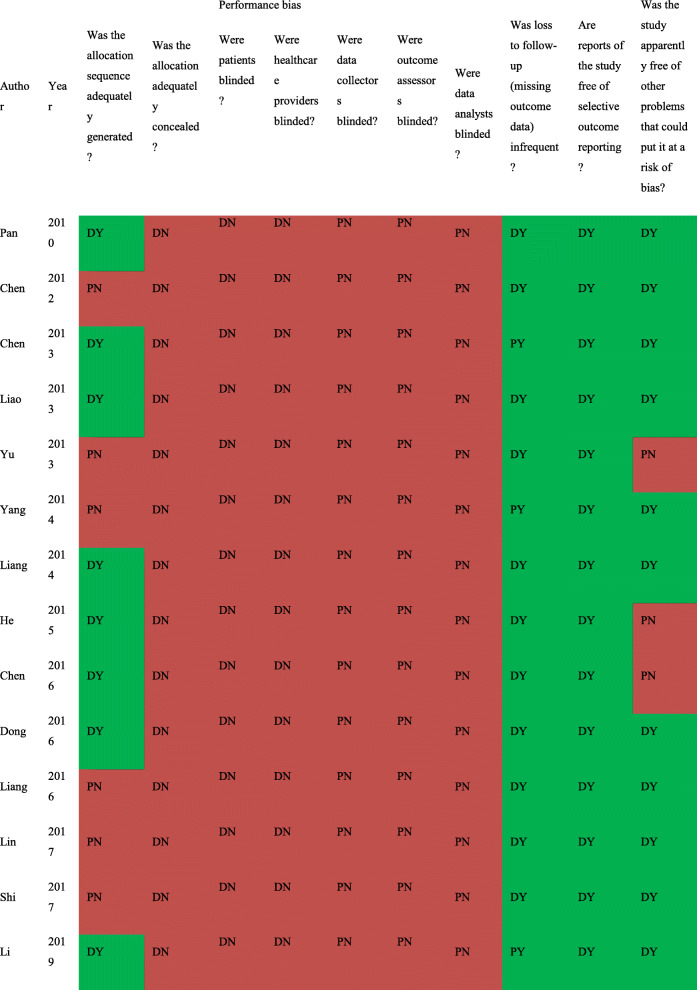
Potential risk of bias of each included studies

*DY = Definitely Yes (Low risk of bias); DN = Definitely No (High risk of bias); PY=Probably Yes; PN=Probably No.

**Table 3:** GRADE evidence profile

Certainty assessment						No. of patients		Effect	Certainty
No. of studies	Risk of bias	Inconsistency	Indirectness	Imprecision	Publication bias	Baduanjin	Control	Absolute(95% CI)	
Cardiovascular morbidity and mortality as possibly influenced by systolic blood pressure									
14 (RCT)	Serious risk of bias ^a^	Serious inconsistency ^b^	Serious indirectness ^c^	No serious imprecision	Undetected	532	526	MD 8.52 **lower** (6.40 to 10.65 lower)	⨁◯◯◯ VERY LOW
Cardiovascular morbidity and mortality as possibly influenced by diastolic blood pressure									
14 (RCT)	Serious risk of bias	Serious inconsistency	Serious indirectness	No serious imprecision	Undetected	532	526	MD 4.65 lower(2.74 to 6.55 lower)	⨁◯◯◯VERY LOW
Cardiovascular morbidity and mortality as possibly influenced by glucose									
3 (RCT)	Serious risk of bias	Serious inconsistency	Serious indirectness	Serious imprecision ^d^	Suspected ^e^	124	124	MD **0.44 lower** (0.21 to 0.67 lower)	⨁◯◯◯ VERY LOW
Cardiovascular morbidity and mortality as possibly influenced by serum total triglyceride									
3 (RCT)	Serious risk of bias	No serious inconsistency	Serious indirectness	Serious imprecision	Suspected	124	124	MD **0.35 lower** (0.07 to 0.64 lower)	⨁◯◯◯ VERY LOW
Cardiovascular morbidity and mortality as possibly influenced by serum total cholesterol									
3 (RCT)	Serious risk of bias	Serious inconsistency	Serious indirectness	Serious imprecision	Suspected	124	124	MD **0.71 lower** (0.21 to 1.21 lower)	⨁◯◯◯ VERY LOW
Cardiovascular morbidity and mortality as possibly influenced by high density lipoprotein cholesterol									
2 (RCT)	Serious risk of bias	No serious inconsistency	Serious indirectness	Serious imprecision	Suspected	54	54	MD **0.29 Higher** (0.09 to 0.48 higher)	⨁◯◯◯ VERY LOW

*CI* Confidence interval, *MD* Mean difference

Explanations

^a^. Blinding cannot be achieved in participants and investigators

^b^. High I square

^c^. Surrogate outcome for cardiovascular morbidity and mortality

^d^. Recommendation would differ if the upper versus the lower boundary of the CI represented the truth

^e^. Only few studies and small in size

### Quantitative analysis

#### SBP

**Fig. 4:**
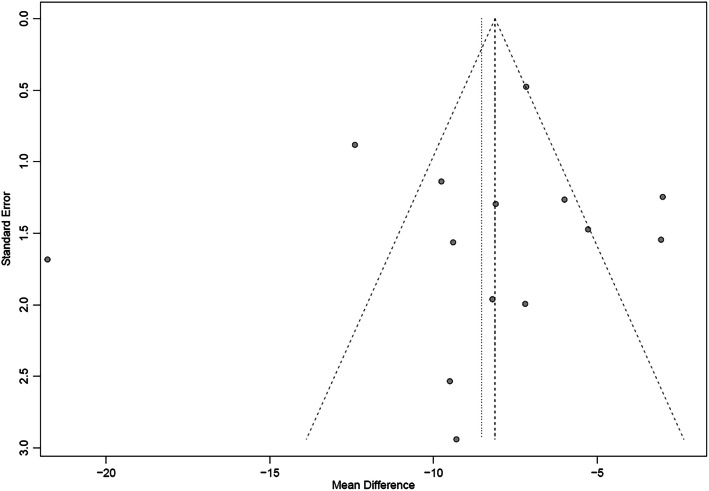
Meta-analysis of SBP including subgroup analysis. SBP: Systolic Blood Pressure

Pooled data from 14 trials provided low certainty evidence that EB might be more effective to lower SBP than control treatments (MD = -8.52. mmHg, 95%CI: [− 10.65, − 6.40], I^2^ = 90%, *P* < 0.01) (Fig. 4). Ten studies showed that EB combined with routine treatment (either antihypertensive drugs or Chinese decoctions or both of them) was more effective than these alternatives alone (MD = -7.24 mmHg, 95%CI: [− 9.60, − 4.89], I^2^ = 85%, P < 0.01). Similar effects were achieved when four studies compare combined effectiveness of EB plus health education versus health education alone (MD = -11.64 mmHg, 95%CI: [− 17.15, − 6.12], I^2^ = 96% *P* < 0.01). When considering effects on patient-important endpoints of cardiovascular morbidity and mortality the evidence is very low certainty (Table 3).

#### DBP

The merged data indicated that EB had a low certainty evidence of being more effective in lowering DBP than the control group (MD = -4.65 mmHg, 95%CI: [− 6.55, − 2.74], I^2^ = 93% *P* < 0.01) (Fig. 2). Subgroup analysis of ten studies illustrated the difference between EB combined with routine treatment and those alternatives alone had statistical significance (MD = -4.08 mmHg, 95%CI: [− 7.13, 1.03], I^2^ = 97% P < 0.01). Compared with health education alone, a combination of EB with health education resulted in a lower DBP (MD = -5.83 mmHg, 95%CI: [− 8.12, − 3.54], I^2^ = 93% P < 0.01). Table 3 also illustrated the certainty of evidence was very low when the effects of DBP was related to cardiovascular morbidity and mortality.

### Subgroup effects

**Fig. 5:**
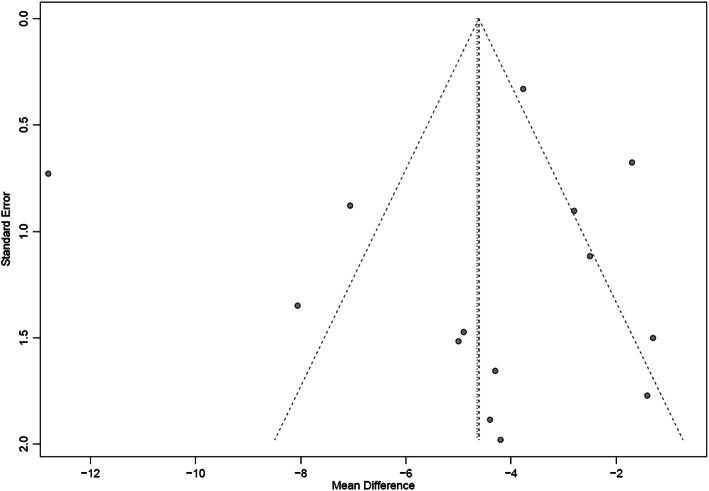
Meta-analysis of DBP including subgroup analysis. DBP: Diastolic Blood Pressure

With respect to the subgroup effects, test of interaction demonstrated that differences between groups could be easily attributed to chance (SBP *P* = 0.15; DBP *P* = 0.37) (Figs. 4 and 5). Based on the five-item guidance (See Additional file 2), the subgroup difference has very low credibility. Besides, we also did meta-regression for the period of intervention and found no statistical significance (*P* = 0.0995).

### Secondary outcomes

Secondary outcomes for these hypertension patients found that EB had statistical significance (MD = -0.44 mmol/L, 95%CI: [− 0.67, − 0.21], I^2^ = 72% *P* < 0.01) (Additional file 3, Figure A3–1) in lowering GLU and of very low certainty evidence. EB was superior to control group (MD = -0.35 mmol/L, 95%CI: [− 0.64, − 0.07], I^2^ = 48% *P* = 0.01) in lowering TG according to our meta-analysis of 3 trials, but also had a very low certainty evidence in the GRADE rating (Additional file 3, Figure A3–2). Three trials reported the effectiveness of EB in lowering TC, and the combined effects indicated that of a very low certainty evidence EB had a better TC compared with control group (MD = -0.71 mmol/L, 95%CI: [− 1.21, − 0.21], I^2^ = 78% *P* < 0.01) (Additional file 3, Figure A3–3). Two trials reported the effectiveness of EB on increasing HDL-C. EB was more effective than control group (MD = 0.29 mmol/L, 95%CI: [0.09, 0.48], I^2^ = 28% P < 0.01) (Additional file 3, Figure A3–4), but it also have a very low certainty evidence according to Table 3. There was only one trial reported LDL-C, of very low certainty evidence. LDL-C could be lowered significantly (MD = -0.59 mmol/L, 95%CI: [− 0.98, − 0.20], *P* = 0.003) in EB group after 6-month’s exercising.

## Discussion

### Main findings

Low certainty of evidence suggested that EB lowers the surrogate outcomes of SBP and DBP; the evidence becomes very low when we consider indirectness with regard to patient-important cardiovascular morbidity and mortality (Table 3). The effect of EB appears similar whether the comparison is of EB plus routine treatment versus routine treatment alone, or EB plus health education versus health education alone (Figs. 4 and 5, Additional file 2). Significant test of interaction was not found in subgroup analyses either of SBP or DBP, so we cannot reject the null hypothesis that claims chance could be totally explained away the subgroup difference. Based on the five-item guidance (See Additional file 2), the subgroup difference could not be proven credible.

As for secondary outcomes, results suggested that EB might exert a positive impact on decreasing GLU, TG, TC and increasing HDL-C, though the evidence of the studies was of very low certainty even without considering the indirectness with respect to major cardiovascular events.

### Strengths and limitations

Strengths of this include a comprehensive search that includes all relevant randomized trials published up to March 30, 2020. The review considered the possibility that the impact of EB was more remarkable when administered with education in comparisons to education alone and found, applying criteria of Sun and his colleagues [26], no suggestion of a subgroup effect. Additionally, we used GRADE as the tool to evaluate the certainty of evidence, and considered certainty both with respect to the surrogate outcomes and, considering indirectness, the certainty with respect to the cardiovascular endpoints of importance to patients.

The review also has limitations. First, heterogeneity for the subgroups was high, and a possible explanation is the clinical heterogeneity due to types of EB (sitting EB, self-made anti-hypertension EB or traditional EB), duration of experiments (from four weeks to one year), age groups, duration of hypertension, and levels of hypertension of patients that limitations in the studies did not allow us to explore. In addition, the definition of routine treatment varied widely within our eligible trials including different kinds of antihypertensive drugs, walking, Chinese herbal decoctions, and some of the studies that failed to point out the detailed routine treatment methods. These differences may have explained heterogeneity, but variability was too great to allow us to explore this possibility with subgroup analysis. Second, every study suffered from high risk of bias (Table 2). For example, no study included blinding as part of the study design. Besides, only Yu’s study [37] which conducted a one-year long follow-up, followed patients for more than 6 months. Consequently, the long-term effectiveness of EB is even less certain that the short-term effectiveness. No trial mentioned adverse events, suggesting a lack of awareness among the investigators regarding collecting safety data for EB interventions. As for the outcome collected, quality of life (QoL) is a commonly used measure of effectiveness, and patient-important for chronic disease, but this endpoint was only reported in 2 trials [30, 31]*,* using SF-36 and self-made simple QoL scale respectively. Therefore, the effect of EB on QoL remains unclear. Although the data did not allow the further subgroup analysis based on QoL, but the low credibility of subgroup effect is clear. Finally, we did not search for trials addressing our secondary outcomes, but only included results from trials of hypertension that also reported on these other outcomes. There may be many other studies of EB focusing on these outcomes that we did not consider.

### Relation to prior work

Previous experiments have shown that many patients with BP levels > 120/80 mmHg are willing to use complementary and alternative medicine (CAM) [41], among which EB, has been the most frequently studied type of Qigong exercise [42]. Compared with previous reviews, we included more studies and more participants. A review [43] evaluated the effects of Baduanjin Qigong for various health benefits in 2017 which included blood pressure as one of the outcomes. The authors reported results similar to ours but presented effects as standardized mean difference (SMD) which is less transparent than the MDs we report. Moreover, they did not rate certainty of evidence using GRADE, nor did they conduct any subgroup analyses.

Another review published in 2015 [13, 44], evaluated the effectiveness of EB primarily on blood pressure and conducted a subgroup analysis between the EB and control groups using three different comparisons. They regarded health education as no intervention while we thought the administration of health education could modify the effect of EB, thus motivating our subgroup analysis. Their findings were similar to ours, but they did not provide a GRADE certainty of evidence rating.

### Implications and future directions

With low requirements for space and weather conditions, EB is easy to learn with soothing actions, and is thus suitable for all age populations. Statistical results illustrated that EB may be effective for the treatment of hypertension, when combined with either routine treatment or health education. However, the evidence for the surrogate outcomes is low certainty (serious limitations in risk of bias and inconsistency) and for cardiovascular morbidity and mortality very low because of indirectness (no study measured cardiovascular outcomes). Moreover, even if EB is effective, there is no standard regarding the appropriate intensity and duration of EB for the improvement of hypertension, and thus optimal administration remains uncertain.

Rigorously designed RCTs that address patient-important outcomes and with longer follow-up duration therefore remain warranted. Such studies should document patient characteristics (age, duration of disease, habits and customs); details of interventions and controls; consider blinding at least of those assessing outcome and data analysts, and possibly through use of an attention placebo the intervention itself; and follow patients for at least one year.

## Conclusions

In summary, EB, as a complementary treatment, may be helpful to control BP, lower blood glucose, improve lipid status, either combined with either routine treatment or health education, and thus possibly influence cardiovascular morbidity and mortality. However, the certainty of current evidence is very low due to high risk of bias, inconsistency, and indirectness.

## Supplementary information

**Additional file 1.** Search strategies. Presents the search strategies used in each database.

**Additional file 2 **Criteria for assessing the credibility of significant subgroup effects. We assessed the credibility of significant subgroup effects (*P* < 0.05) using a five-criteria list.

**Additional file 3.** Meta-analysis of Secondary outcomes including glucose, serum total triglyceride, serum total cholesterol, and high density lipoprotein cholesterol.

## Data Availability

The data used to support the findings of this study are available from the corresponding author upon request.

## Abbreviations

EB: Eight Brocades, Baduanjin
RCTs: randomized controlled trials
MD: mean difference
SBP: systolic blood pressure
DBP: diastolic blood pressure
HBP: High blood pressure
GLU: Glucose
TG: Serum Total Triglyceride
TC: Serum Total Cholesterol
HDL-C: High Density Lipoprotein Cholesterol
LDL-C: Low Density Lipoprotein Cholesterol
ROB: risk of bias
BP: blood pressure
WHO: world health organization
QoL: quality of life
CAM: complementary and alternative medicine
SMD: standardized mean difference

## References

[1] Benjamin E, Virani S, Callaway C, Chamberlain A, Chang A, Cheng S, Chiuve S, Cushman M, Delling F, Deo RJC. Heart Disease and Stroke Statistics-2018 Update: A Report From the American Heart Association. Circulation. 2018;137(12):e67. 10.1161/CIR.0000000000000558 29386200

[2] Mills KT, Stefanescu A, He J (2020). The global epidemiology of hypertension. Nat Rev Nephrol.

[3] World Health O. A global brief on hypertension: silent killer, global public health crisis: World Health day 2013: World Health Organization; 2013.

[4] Kochanek KD, Murphy SL, Xu J, Tejada-Vera B (2016). Deaths: final data for 2014. Natl Vital Stat Rep.

[5] Ford ES (2011). Trends in mortality from all causes and cardiovascular disease among hypertensive and nonhypertensive adults in the United States. Circulation.

[6] Campbell NRC, Lackland DT, Lisheng L, Niebylski ML, Nilsson PM, Zhang XH (2015). Using the global burden of disease study to assist development of nation-specific fact sheets to promote prevention and control of hypertension and reduction in dietary salt: a resource from the World hypertension league. J Clin Hypertension.

[7] Ogihara T, Kikuchi K, Matsuoka H, Fujita T, Higaki J, Horiuchi M, Imai Y, Imaizumi T, Ito S, Iwao HJHR. The Japanese Society of Hypertension guidelines for the management of hypertension (JSH 2009). Hypertens Res. 2009;32(1):3–107. 19300436

[8] Laurent S (2017). Antihypertensive drugs. Pharmacol Res.

[9] Whelton PK, Carey RM, Aronow WS, Casey DE, Collins KJ, Dennison Himmelfarb C, DePalma SM, Gidding S, Jamerson KA, Jones DW (2018). 2017 ACC/AHA/AAPA/ABC/ACPM/AGS/APhA/ASH/ASPC/NMA/PCNA guideline for the prevention, detection, evaluation, and Management of High Blood Pressure in adults: executive summary: a report of the American College of Cardiology/American Heart Association task force on clinical practice guidelines. Hypertension (Dallas, Tex : 1979).

[10] Rapsomaniki E, Timmis A, George J, Pujades-Rodriguez M, Shah AD, Denaxas S, White IR, Caulfield MJ, Deanfield JE, Smeeth L (2014). Blood pressure and incidence of twelve cardiovascular diseases: lifetime risks, healthy life-years lost, and age-specific associations in 1· 25 million people. Lancet.

[11] Joint Committee for Guideline Revision. 2018 Chinese guidelines for prevention and treatment of hypertension-a report of the revision Committee of Chinese Guidelines for prevention and treatment of hypertension. J Geriatric Cardiol. 2019;16(3):182–241. 10.11909/j.issn.1671-5411.2019.03.014 PMC6500570 31080465

[12] Cornelissen VA, Smart NA (2013). Exercise training for blood pressure: a systematic review and meta-analysis. J Am Heart Assoc.

[13] Zou L, Sasaki JE, Wang H, Xiao Z, Fang Q, Zhang M. A systematic review and meta-analysis of baduanjin qigong for health benefits: randomized controlled trials. Evid Based Complement Alternat Med. 2017;2017:4548706. 10.1155/2017/4548706 PMC5359459 28367223

[14] Zheng G, Chen B, Fang Q, Lin Q, Tao J, Chen L (2019). Baduanjin exercise intervention for community adults at risk of ischamic stroke: a randomized controlled trial. Sci Rep.

[15] Ye J, Simpson MW, Liu Y, Lin W, Zhong W, Cai S, Zou L. The effects of Baduanjin qigong on postural stability, proprioception, and symptoms of patients with knee osteoarthritis: a randomized controlled trial. Front Med. 2019;6:307. 10.3389/fmed.2019.00307 PMC6966958 31998728

[16] Wen J, Lin T, Jiang C, Peng R, Wu W (2017). Effect of Baduanjin exercises on elevated blood lipid levels of middle-aged and elderly individuals: protocol for a systematic review and meta-analysis of randomised controlled trials. BMJ Open.

[17] Liu T, Bai S, Zhang RC (2018). Effects of Health Qigong Baduanjin on diabetes related indexes in middle-aged obese women. Zhongguo Ying Yong Sheng Li Xue Za Zhi.

[18] Liu S-J, Ren Z, Wang L, Wei G-X, Zou L (2018). Mind–body (Baduanjin) exercise prescription for chronic obstructive pulmonary disease: a systematic review with meta-analysis. Int J Environ Res Public Health.

[19] Xiao C, Yang Y, Zhuang Y (2016). Effect of health qigong Ba Duan Jin on blood pressure of individuals with essential hypertension. J Am Geriatr Soc.

[20] Writing Group of 2010 Chinese Guidelines for the Management of Hypertension. 2010 Chinese guidelines for the management of hypertension. Chin J Cardiol. 2011;39(7):579–615. 22088239

[21] Chalmers J, MacMahon S, Mancia G, Whitworth J, Beilin L, Hansson L, Neal B, Rodgers A, Ni Mhurchu C, Clark T (1999). 1999 World Health Organization-International Society of Hypertension Guidelines for the management of hypertension. Guidelines sub-committee of the World Health Organization. Clin Exp Hypertens.

[22] Akl EA, Sun X, Busse JW, Johnston BC, Briel M, Mulla S, You JJ, Bassler D, Lamontagne F, Vera C (2012). Specific instructions for estimating unclearly reported blinding status in randomized trials were reliable and valid. J Clin Epidemiol.

[23] Higgins JPT, Altman DG, Gøtzsche PC, Jüni P, Moher D, Oxman AD, Savović J, Schulz KF, Weeks L, Sterne JAC (2011). The Cochrane Collaboration’s tool for assessing risk of bias in randomised trials. BMJ.

[24] Modifcation of Cochrane Tool to assess risk of bias in randomized trials. https://www.evidencepartners.com/wp-content/uploads/2017/09/Tool-to-Assess-Risk-of-Bias-in-Randomized-Controlled-Trials.pdf. Accessed 5 Apr 2020.

[25] Guyatt G, Oxman AD, Akl EA, Kunz R, Vist G, Brozek J, Norris S, Falck-Ytter Y, Glasziou P, Debeer H (2011). GRADE guidelines: 1. Introduction—GRADE evidence profiles and summary of findings tables. J Clin Epidemiol.

[26] Sun X, Ioannidis JPA, Agoritsas T, Alba AC, Guyatt G (2014). How to use a subgroup analysis: users’ guide to the medical literature. Jama.

[27] Chen H, Zhou Y (2012). Effect of Baduanjin on blood pressure and serum high sensitivity C reactive protein in patients with essential hypertension. Chin J Rehabil Med.

[28] Dong C, Zhang Y (2016). Application of “Jiang Ya Ba Duan Jin”on grade I primary hypertension control of middle-aged patients. Nursing Journal of Chinese People's Liberation Army.

[29] He X (2015). Rehabilitation therapeutic effect of Baduanjin training in aged patients with hypertension. Chin J Cardiovasc Rehabil Med.

[30] Huiyang. Effect of Baduanjin on Cardiovascular Autonomic Nerve Regulation and on the Quality of Life in Patients with Hypertension: Hebei United University; 2014.

[31] Liang H, Huang C, Li D (2016). Effect of Baduanjing on blood pressure and quality of life in patients with isolated systolic hypertension. Chin Manipul Rehabil Med.

[32] Liang Y, Liao S, Han C, Wang H, Peng Y (2014). Effect of Baduanjing intervention on blood pressure and blood lipids in patients with essential hypertension. Henan Tradit Chin Med.

[33] Liao S, Liang Y, Xia L, Tan Y (2013). The influence of traditional Chinese medicine body-building gong Baduanjin on patients with metabolic syndrome. Modern J Integr Tradit Chin West Med.

[34] Lin Q, Yan X. Promoting Effect of Fitness Baduanjin on Rehabilitation of Elderly Patients with Hypertension. Chin J Gerontol. 2017;037(012):3024–26.

[35] Pan H, Feng Y (2010). Clinical observation of rehabilitation therapy with Health qigong Ba Duan Jin on grade 1 hypertension of old patients. J Nanjing Sport Inst (Natural Science).

[36] Shi Z, Miao Z (2017). Treatment of grade 1 hypertension by traditional Chinese medicine physical therapy. Chin Manipul Rehabil Med.

[37] Yu H (2013). Clinical observation of Baduanjin therapy on 104 patients with hypertension and obesity. Chin J Clin.

[38] Chen L. Application of Baduanjin in rehabilitation nursing of elderly patients with hypertension. Med Front. 2016;6(022):340–1.

[39] Chen Q (2013). Study on the mechanism of Baduanjin's antihypertensive effect on grade 1 hypertension from vascular endothelial function.

[40] Li WH, Wu ZF, Jing CX, Pan HS. The effect of Baduanjin on blood glucose and blood pressure in pre diabetes patients with mild hypertension. New Chin Med. 2019;7(89):291–4.

[41] Brook RD, Appel LJ, Rubenfire M, Ogedegbe G, Bisognano JD, Elliott WJ, Fuchs FD, Hughes JW, Lackland DT, Staffileno BA (2013). Beyond medications and diet: alternative approaches to lowering blood pressure: a scientific statement from the american heart association. Hypertension.

[42] Zhang YP, Hu RX, Han M, Lai BY, Liang SB, Chen BJ, Robinson N, Chen K, Liu JP. Evidence Base of Clinical Studies on Qi Gong: A Bibliometric Analysis. Complement Ther Med. 2020;50:102392. 10.1016/j.ctim.2020.102392. 10.1016/j.ctim.2020.102392 32444061

[43] Zou L, SasaKi JE, Wang H, Xiao Z, Fang Q, Zhang M. A Systematic Review and Meta-Analysis Baduanjin Qigong for Health Benefits: Randomized Controlled Trials. Evidence-based complementary and alternative medicine: eCAM. 2017. p. 4548706. 10.1155/2017/4548706. 10.1155/2017/4548706 PMC5359459 28367223

[44] Xiong X, Wang P, Li S, Zhang Y, Li X (2015). Effect of Baduanjin exercise for hypertension: a systematic review and meta-analysis of randomized controlled trials. Maturitas.

