# Supplementary material for: The effectiveness of Baduanjin exercise for hypertension: a systematic review and meta-analysis of randomized controlled trials
Source: BMC Complement Med Ther. 2020 Oct 8;20:304. doi: 10.1186/s12906-020-03098-w (PMC7545896; doi:10.1186/s12906-020-03098-w)
Supplement: Supplementary file 1 — Additional file 1. Search strategies. Presents the search strategies used in each database. [file 12906_2020_3098_MOESM1_ESM.docx]

**Additional file 1. Search strategies**

| **Database** | **Search strategies** |
| --- | --- |
| CNKI | SU = '高血压'*'八段锦' |
| VIP | M=八段锦 AND M=高血压 |
| Wanfang Data | 主题:八段锦 AND 主题:高血压 |
| CBM | ("Baduanjin"[ All Fields: Smart]) AND ("hypertension"[ All Fields: Smart]) |
| PubMed | ((baduanjin[All Fields] OR (eight[All Fields] AND trigrams[All Fields] AND ("boxing"[MeSH Terms] OR "boxing"[All Fields]))) OR (eight[All Fields] AND brocades[All Fields])) AND ("hypertension"[MeSH Terms] OR "hypertension"[All Fields]) |
| Web of Science | TS=(Baduanjin OR Eight trigrams boxing OR Eight brocade) AND TS=hypertension |
| The Cochrane Library | #1 (Baduanjin):ti,ab,kw OR (Eight trigrams boxing):ti,ab,kw OR (Eight brocade):ti,ab,kw (Word variations have been searched)  #2 (hypertension):ti,ab,kw (Word variations have been searched)  #3 #1 AND #2 |
| Scopus | Baduanjin OR (Eight AND trigram AND boxing) OR (Eight AND brocade) AND hypertension |
| *’SU’=subject;’ TI’=title; ’M’=keyword or title; ‘[Common fields: Smart]’=To achieve extended retrieval of search terms and their synonyms (including key words) with Chinese titles, abstracts, keywords and key words; Topic terms in Web of Science indicates the following fields: title, abstract, author keywords and keywords plus. | |
