# Supplementary material for: The effectiveness of Baduanjin exercise for hypertension: a systematic review and meta-analysis of randomized controlled trials
Source: BMC Complement Med Ther. 2020 Oct 8;20:304. doi: 10.1186/s12906-020-03098-w (PMC7545896; doi:10.1186/s12906-020-03098-w)
Supplement: Supplementary file 2 — Additional file 2 Criteria for assessing the credibility of significant subgroup effects. We assessed the credibility of significant subgroup effects (P < 0.05) using a five-criteria list. [file 12906_2020_3098_MOESM2_ESM.docx]

**Additional file 2. Criteria for assessing the credibility of significant subgroup effects**

| **Criteria** | **Answer** | **Explanations** |
| --- | --- | --- |
| 1. Can Chance Explain the Subgroup Difference? | Yes | P>0.10 for test of interaction |
| 2. Is the subgroup difference consistent across studies? | No | Large heterogeneity with each subgroup. |
| 3. Was the subgroup difference one of a small number of a priori hypotheses in which the direction was accurately prespecified? | Yes | A prior hypothesis including the effects’ direction was defined in advance. |
| 4. Is there a strong preexisting biological rationale supporting the apparent subgroup effect? | No | We presume health education, as a non-medicine intervention, will have a relatively smaller effects compared with routine treatment. However, there is still lack of biological evidence. |
| 5. Is the subgroup difference suggested by comparisons within rather than between studies? | No | This was a between-study comparison. Co-intervention, length of intervention and risk of bias differ between studies. |
