# Supplementary material for: The effectiveness of Baduanjin exercise for hypertension: a systematic review and meta-analysis of randomized controlled trials
Source: BMC Complement Med Ther. 2020 Oct 8;20:304. doi: 10.1186/s12906-020-03098-w (PMC7545896; doi:10.1186/s12906-020-03098-w)
Supplement: Supplementary file 3 — Additional file 3. Meta-analysis of Secondary outcomes including glucose, serum total triglyceride, serum total cholesterol, and high density lipoprotein cholesterol. [file 12906_2020_3098_MOESM3_ESM.docx]

**Additional file 3. Meta-analysis of Secondary outcomes**


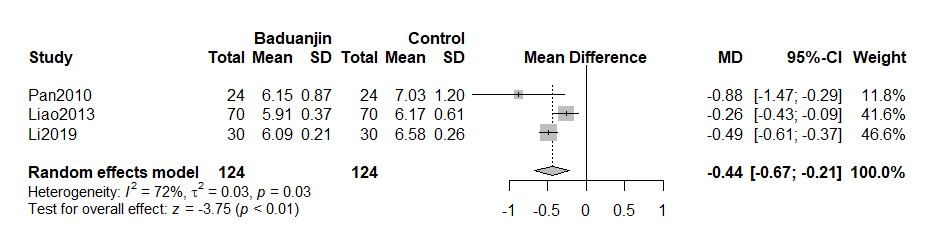


**Figure A3-1** Meta-analysis of GLU. GLU:Glucose


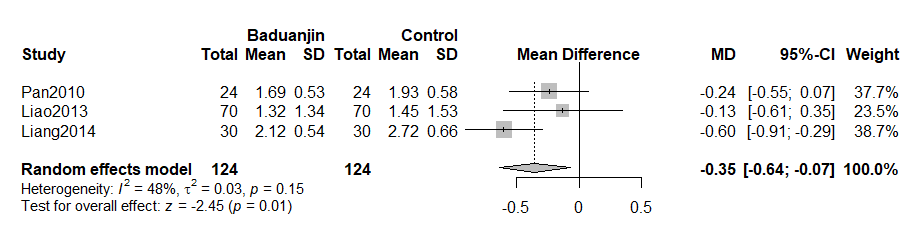


**Figure A3-2** Meta-analysis of TG. TG: Serum Total Triglyceride


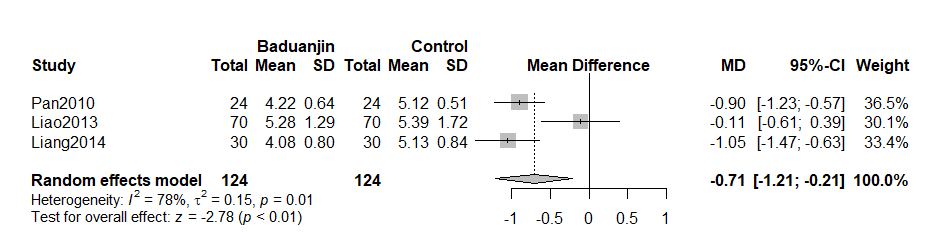
 **Figure A3-3** Meta-analysis of TC. TC: Serum Total Cholesterol


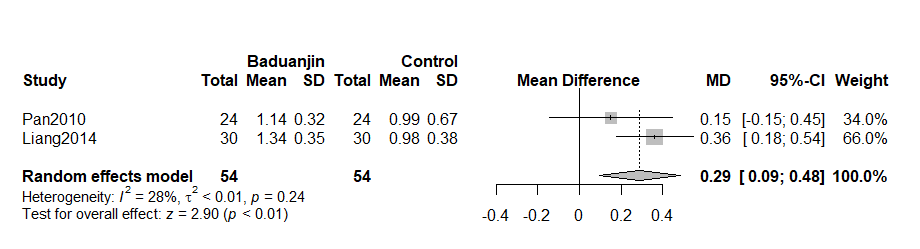


**Figure A3-4** Meta-analysis of HDL-C. HDL-C: High Density Lipoprotein Cholesterol
